# Supplementary material for: Performance analysis of conventional and AI-based variant callers using short and long reads
Source: BMC Bioinformatics. 2023 Dec 14;24:472. doi: 10.1186/s12859-023-05596-3 (PMC10720095; doi:10.1186/s12859-023-05596-3)
Supplement: Supplementary file 1 — Additional file 1: Table S1. Detailed results for performance analysis of calling variants from three samples using five variant calling tools with different sequencing technologies (Illuina, Pacbio HiFi, and ONT) in terms of accuracy metrics (precision, recall and F1-score), running time, and memory. [file 12859_2023_5596_MOESM1_ESM.pdf]

Table S1. Detailed results for performance analysis of calling variants from three samples using five variant calling tools with different sequencing technologies (Illuina, Pacbio HiFi, and ONT) in terms of accuracy metrics (precision, recall and F1-score), running time, and memory**.**

			AI-based												Conventional			
			DNAscope			DeepVariant			Platypus			BCFTools			GATK4			
			Illumina	PacBio HiFi	ONT	Illumina	PacBio HiFi	ONT	Illumina	PacBio HiFi	ONT	Illumina	PacBio HiFi	ONT		Illumina	PacBio HiFi	ONT
HG003	INDEL	Precsion	0.5878	0.9955		0.8449	0.9949	0.1035	0.9257			0.8775	0.8300	0.0000		0.5842	0.7422	
		Recall	0.8071	0.9954		0.7317	0.9949	0.4232	0.5471			0.6925	0.8829	0.0004		0.7110	0.8969	
		F1score	0.6802	0.9955		0.7842	0.9949	0.1663	0.6878			0.7741	0.8557	0.0000		0.6414	0.8123	
	SNP	Precsion	0.9620	0.9993		0.9867	0.9995	0.9968	0.9817			0.9861	0.9894	0.8759		0.9600	0.9955	
		Recall	0.9418	0.9994		0.9213	0.9992	0.9769	0.8018			0.9032	0.9964	0.9445		0.9129	0.9960	
		F1score	0.9518	0.9993		0.9528	0.9994	0.9868	0.8827			0.9428	0.9929	0.9089		0.9358	0.9958	
		Time (hours)	3.4000	11.8333		5.1833	35.6833	105.2167	0.2580			2.5500	36.7500	7.9833		36.6000	100.7667	
		Memory (GB)	3.3100	10.7100		17.3300	51.0800	33.8500	2.8500			0.4938	6.9500	2.1200	Round 1	20.6500	42.8000	
															Round 2	27.5200	29.2200	
HG006	INDEL	Precsion	0.3588	0.9969		0.8770	0.9965		0.9410			0.9076	0.8553			0.6127	0.7446	
		Recall	0.8557	0.9968		0.7940	0.9969		0.6506			0.7775	0.9125			0.7908	0.9233	
		F1score	0.5056	0.9968		0.8334	0.9967		0.7693			0.8375	0.8830			0.6905	0.8244	
	SNP	Precsion	0.9328	0.9994		0.9917	0.9996		0.9870			0.9898	0.9903			0.9675	0.9957	
		Recall	0.9623	0.9995	N/A	0.9419	0.9994		0.8799	N/A	N/A	0.9452	0.9974			0.9423	0.9961	N/A
		F1score	0.9473	0.9994		0.9662	0.9995		0.9304			0.9670	0.9939			0.9547	0.9959	
		Time (hours)	5.2333	10.7333		4.1667	24.0833		0.3833			2.9500	40.3333			50.1833	96.8500	
		Memory (GB)	4.1500	13.3000		16.4600	53.1500		2.6500			0.4938	7.9200		Round 1	24.9000	23.9000	
								N/A						N/A	Round 2	23.8700	28.1100	
HG007	INDEL	Precsion	0.3968	0.9953		0.8728	0.9924		0.9394			0.9050	0.7360			0.6376	0.5625	
		Recall	0.8452	0.9950		0.7814	0.9933		0.6374			0.7576	0.8941			0.7718	0.9074	
		F1score	0.5400	0.9952		0.8246	0.9928		0.7595			0.8247	0.8074			0.6983	0.6945	
	SNP	Precsion	0.9395	0.9993		0.9900	0.9995		0.9861			0.9891	0.9898			0.9692	0.9961	
		Recall	0.9565	0.9993		0.9375	0.9992		0.8668			0.9334	0.9970			0.9335	0.9957	
		F1score	0.9479	0.9993		0.9631	0.9993		0.9226			0.9604	0.9934			0.9511	0.9959	
		Time (hours)	4.5500	12.4167		4.3000	19.9500		0.3833			2.8000	39.8500			45.7833	110.8667	
		Memory (GB)	3.8500	9.4000		16.5600	48.4500		2.6700			0.4943	12.2300		Round1	24.9900	36.3500	
															Round2	29.2400	33.3600	
