# Supplementary material for: Performance analysis of conventional and AI-based variant callers using short and long reads
Source: BMC Bioinformatics. 2023 Dec 14;24:472. doi: 10.1186/s12859-023-05596-3 (PMC10720095; doi:10.1186/s12859-023-05596-3)
Supplement: Supplementary file 2 — Additional file 2: Fig. S1. Venn diagrams representing the degree of overlap among variants called using five variant callers and Illumina data compared to the GIAB truth sets. A, B, and C represent SNVs, while D, E, and F represent INDELs for HG003, HG006, and HG007, respectively. Fig. S2. Venn diagrams representing the degree of overlap among variants called using four variant callers and PacBio HiFi data compared to the GIAB truth sets. A, B, and C represent SNVs, while D, E, and F represent INDELs for HG003, HG006, and HG007, respectively. Fig. S3. Venn diagrams representing the degree of overlap among variants called using two variant callers and ONT data compared to the GIAB truth sets. A represents SNVs, while B represents INDELs for HG003. [file 12859_2023_5596_MOESM2_ESM.pdf]

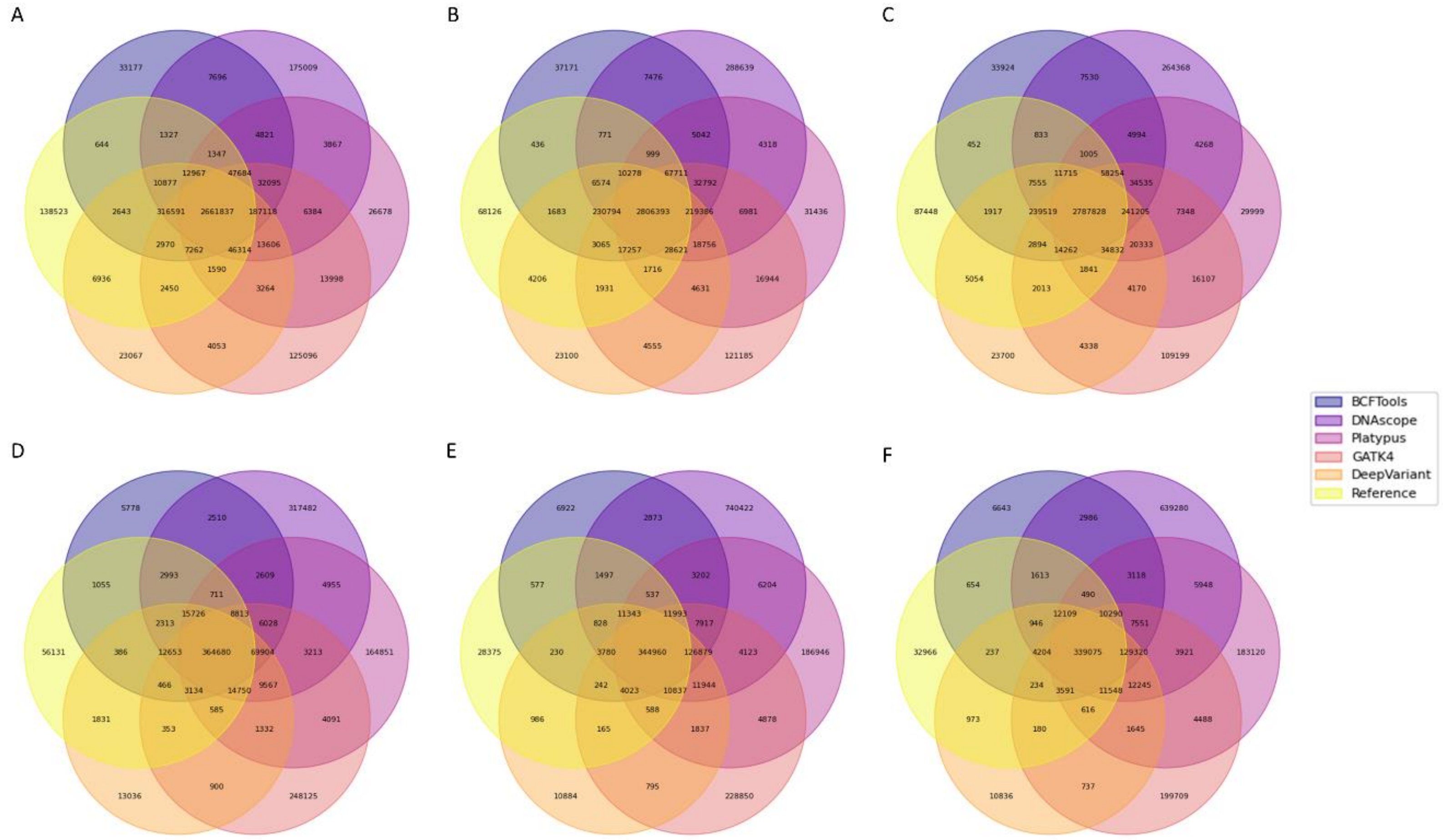

Figure S1. Venn diagrams representing the degree of overlap among variants called using five variant callers and Illumina data compared to the GIAB truth sets. A, B, and C represent SNVs, while D, E, and F represent INDELs for HG003, HG006, and HG007, respectively.

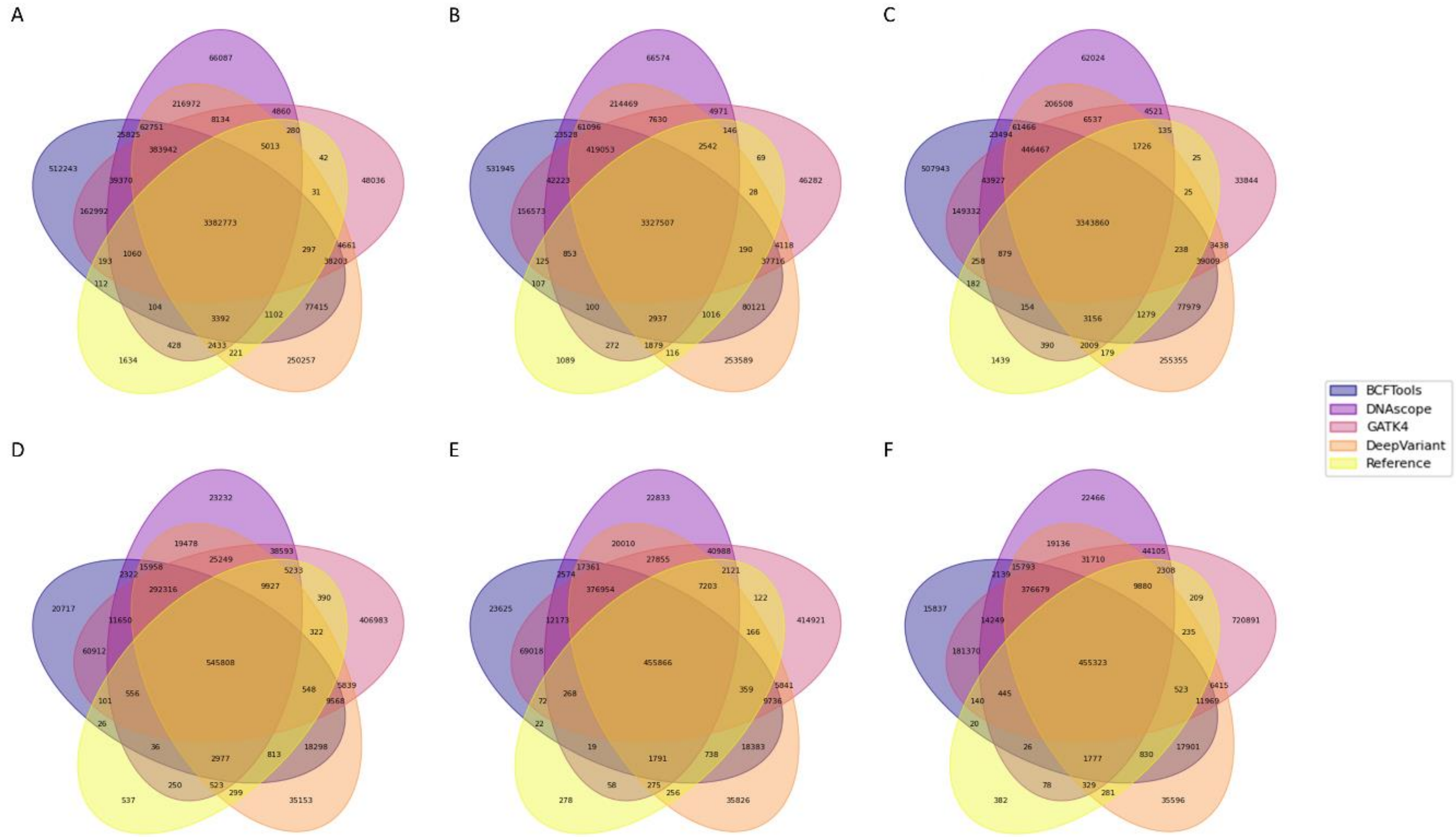

Figure S2. Venn diagrams representing the degree of overlap among variants called using four variant callers and PacBio HiFi data compared to the GIAB truth sets. A, B, and C represent SNVs, while D, E, and F represent INDELs for HG003, HG006, and HG007, respectively.

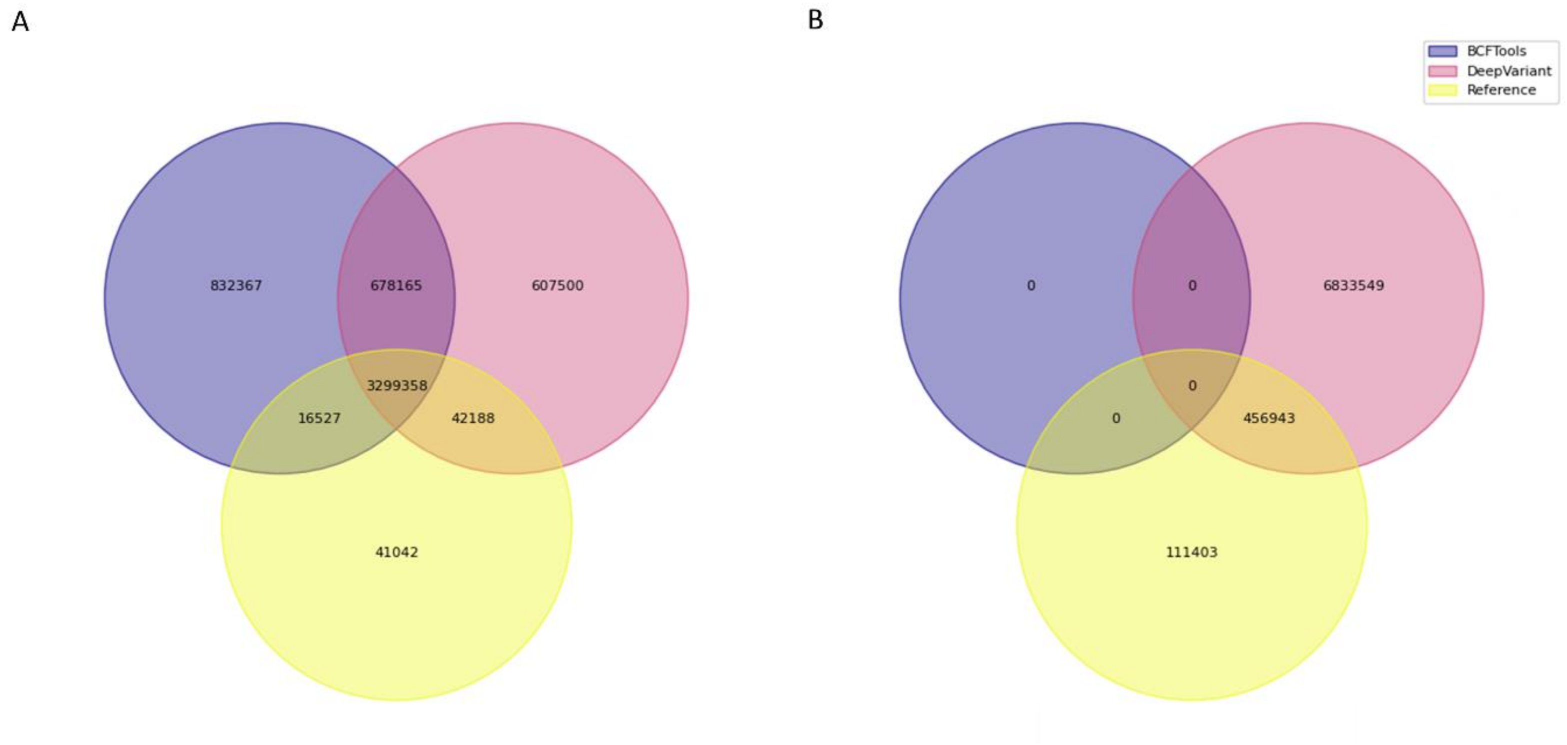

Figure S3. Venn diagrams representing the degree of overlap among variants called using two variant callers and ONT data compared to the GIAB truth sets. A represents SNVs, while B represents INDELs for HG003.
